# Mathematical modeling of a multi-product EMQ model with an enhanced end items issuing policy and failures in rework

**DOI:** 10.1186/s40064-015-1487-4

**Published:** 2015-11-05

**Authors:** Yuan-Shyi Peter Chiu, Peng-Cheng Sung, Singa Wang Chiu, Chung-Li Chou

**Affiliations:** Department of Industrial Engineering and Management, Chaoyang University of Technology, Taichung, 413 Taiwan; Department of Business Administration, Chaoyang University of Technology, Taichung, 413 Taiwan

**Keywords:** Mathematical modeling, Optimization, Multi-product system, Economic manufacturing quantity, Multi-delivery, Rework failures

## Abstract

This study uses mathematical modeling to examine a multi-product economic manufacturing quantity (EMQ) model with an enhanced end items issuing policy and rework failures. We assume that a multi-product EMQ model randomly generates nonconforming items. All of the defective are reworked, but a certain portion fails and becomes scraps. When rework process ends and the entire lot of each product is quality assured, a cost reduction n + 1 end items issuing policy is used to transport finished items of each product. As a result, a closed-form optimal production cycle time is obtained. A numerical example demonstrates the practical usage of our result and confirms a significant savings in stock holding and overall production costs as compared to that of a prior work (Chiu et al. in J Sci Ind Res India, 72:435–440  2013) in the literature.

## Background

Mathematical modeling is used in this study to examine a multi-product EMQ model with rework failures and an enhanced cost reduction end items issuing policy. The EMQ model made use of a mathematical technique to balance the setup and holding costs incurred in a production cycle, and derive most economic manufacturing quantity that minimizes the long run average system costs per unit time (Taft 1918). The assumption of traditional EMQ model includes a perfect manufacturing process for a single product, and a continuous finished product distribution policy. Although the assumptions of the EMQ model are simple and somehow unrealistic, its concept along with solution procedure has since been extensively applied to the fields of inventory control and production management (Hadley and Whitin 1963; Silver et al. 1998; Nahmias 2009; Battini et al. 2010a; Andriolo et al. 2014; Azzi et al. 2014; Glock et al. 2014). In order to increase machine utilization, the vendors in manufacturing sector often fabricate multiple products in sequence on a single machine. Rosenblatt and Finger (1983) considered a single machine multi-item production problem, whereas the machine was an electrochemical machining system, and its outputs are impact sockets of different sizes for power wrenches. They used a grouping procedure for various different products along with a modified version of an existing algorithm to confirm that the cycle times are the multiples of the shortest cycle time. Federgruen and Katalan (1998) examined stochastic economic batch scheduling problems with periodic base-stock policies, where all products are fabricated according to a given periodic item-order. They proposed some effective heuristics to minimize system-wide costs for such a periodic item-sequence production. Muramatsu et al. (2013) studied a multi-item multi-process dynamic lot size scheduling problem with setup time and general product structure. Various heterogeneous decision features such as lot sizing, lot sequencing, dispatching are considered. A near-optimal solution procedure was proposed to determine the decision features involved in this problem simultaneously. Caggiano et al. (2009) proposed a method for computing the channel fill rates in a multi-product, multi-echelon service parts distribution system. A simulation approach was employed to study multi-item three-echelon production- distribution system. They showed that the estimation errors are insignificant over a wide range of base stock level vectors and they also presented an enhanced approximation method to the problem. Jodlbauer and Reitner (2012) investigated a stochastic make-to-order multi-product manufacturing system under a common cycle policy. Effects of the safety stock, demand, cycle time, and setup time on the service level and on the total system costs were investigated. Papers that related to various aspects of planning and optimization issues on multi-item production can also be referred to Lin et al. (2014); Wu et al. (2014).

In real manufacturing environments, due to unpredictable factors production of defective products is inevitable. Sometimes, certain portion of nonconforming products can be reworked and repaired with additional repair expense. Agnihothri and Kenett (1995) studied the effects of defects on various system performance measures for a manufacturing process with rework. They offered management guidelines for allocating additional budget in process improvement for continuously increasing yield rate, identifying potential bottlenecks under increasing workloads, and providing extra resources to release bottlenecks. Extra studies that addressed various aspects of specific features including imperfect quality production and the rework processes can also be found elsewhere (Grosfeld-Nir and Gerchak 2002; Biswas and Sarker 2008; Battini et al. 2010b; Glock 2011a, 2012a; Chiu and Chang 2014; Wee et al. 2014; Murugan and Selladurai 2014; Hishamuddin et al. 2014; Battini et al. 2014; Chiu et al. 2014). The conventional EMQ model assumes a simplified ‘continuous product issuing policy’. However, in real supply chains environments multiple or periodic product delivery policy is commonly adopted. Banerjee (1986) examined a joint economic lot-size model for vendor and buyer, with the focus on minimizing the total joint relevant cost. He concluded that a jointly optimal ordering policy along with an appropriate price adjustment, could be economically beneficial for both buyer and vendor. Swenseth and Godfrey (2002) revealed that the freight rate functions can be combined into inventory replenishment decisions without lowering the accuracy of decisions, nor will these functions increase the complexity of the decision making process. Abdul-Jalbar et al. (2008) studied a one-vendor multi-buyer multi- echelon finite production rate system. The objectives of their study are to determine the optimal production and shipment schedule, and the most economic order size for buyers so that average total cost per unit time can be minimized. In a recent study, Chiu et al. (2013) determined optimal common production cycle time for a multi-item production system with discontinuous *n* fixed quantity multiple delivery policy and rework failures. Mathematical modeling and optimization techniques are employed in their study to solve the problem. As a result, a closed-form optimal common cycle time that minimizes the expected system costs is obtained. Effect of rework failure on the optimal cycle time was investigated through a numerical example. Additional studies that addressed various aspects of periodic or multi-delivery issues of vendor–buyer integrated systems can be referred to (Glock 2011b, 2012b; Katsaliaki et al. 2014; Safaei 2014; Rodger 2014; Sana et al. 2014).

For the purpose of lowering producer’s inventory holding cost as well as minimizing the expected overall system cost, we extend Chiu et al.’s (2013) work by replacing their *n* fixed quantity end items delivery policy with an enhanced *n* + 1 issuing policy. Under the new policy, one extra delivery of end items is made in producer’s production uptime to satisfy customers’ demands during production uptime and rework time. Then, upon completion of the rework, additional *n* installments (fixed quantity) of end items are shipped at a fixed time interval. The objectives of this study are to determine optimal common cycle time that minimizes the long-run average system cost per unit time, and investigate effects of random defective rate, rework failures, and the enhanced end items issuing policy on the optimal operating cycle time as well as on the expected system costs per unit time.

## Problem description and mathematical modeling

**Fig. 1:**
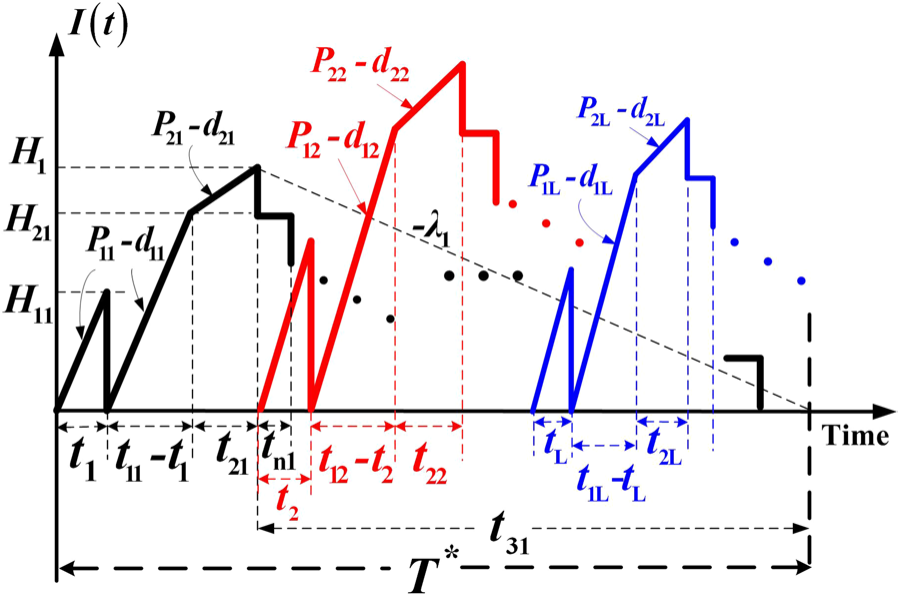
On-hand inventory of perfect quality items in the proposed multi-item production system with rework failures and a cost reduction distribution policy

We use mathematical modeling to examine a multi-product EMQ system with a cost reduction multi-distribution policy and rework failures. Consider there are *L* products to be fabricated in sequence on a single machine and there is a *x*_i_ portion of nonconforming items being randomly produced at a rate *d*_1i_ during the production of product *i* (where $i = 1,{ 2}, \ldots ,L$). All items are screened and cost of quality inspection is included in unit production cost *C*_*i*_. Under the normal operation (which shortage are not permitted), the constant production rate *P*_1i_ for product *i* must satisfies $(P_{{ 1 {\text{i}}}} - d_{{ 1 {\text{i}}}} - \lambda_{\text{i}} ) > 0$, where *λ*_i_ denotes demand rate for product *i* per year, and *d*_1i_ = *x*_i_*P*_1i_. All nonconforming items are reworked at a rate of *P*_2i_ each cycle immediately following the end of regular production process. The additional rework cost is *C*_Ri_ per item. A failure-in-rework rate φ_i_ exists during the reworking process, the production rate of scrap items during rework *d*_2i_ = φ_i_*P*_2i_, and those that fail in repair are discarded at a unit disposal cost *C*_Si_. A specific *n* + 1 multi-shipment policy is proposed in an attempt to reduce the vendor’s stock holding cost as compared to *n* multi-delivery policy used in Chiu et al. (2013). Under the proposed *n* + 1 delivery policy, the purpose of the first shipment of finished goods is to meet buyers’ product demands during vendor’s uptime and reworking time. Then upon completion of rework process, *n* fixed quantity installments of the end items are distributed to buyers at a fixed time interval *t*_n_ (see Fig. 1).

**Fig. 2:**
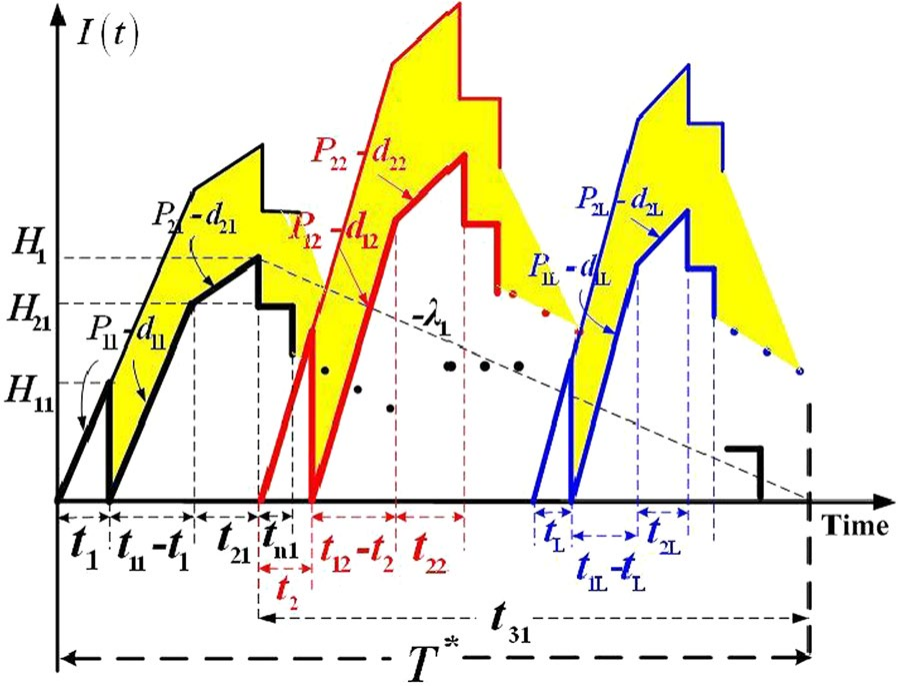
Expected reduction in producer’s inventory holding costs (*yellow shaded area*) for each product *i* in the proposed model in comparison with that in Chiu et al. (2013)

Figure 2 depicts producer’s on-hand inventory level of perfect quality items of product *i* in the proposed *n* + 1 delivery model (in blue lines) and the expected reduction in vendor’s stock holding costs (yellow shaded area) in comparison with that of Chiu et al. (2013). Cost related variables used in our analysis comprise the setup cost *K*_*i*_ per cycle, unit holding cost *h*_*i*_, unit holding cost *h*_1*i*_ during rework, the fixed shipping cost *K*_1*i*_ for product *i* per delivery, and unit shipping cost *C*_T*i*_ for each product *i*. Additional variables also include the following: *T* = common production cycle length, our decision variable, *Q*_*i*_ = batch size per cycle for product *i*, *n* = number of installments (fixed quantity) of the finished lot to be shipped to buyers per cycle, *H*_1*i*_ = on-hand inventory in units of product *i* for meeting buyer’s demand during uptime *t*_1*i*_ and reworking time *t*_2*i*_, *H*_2*i*_ = maximum level of on-hand inventory of product *i* when the regular production ends, *H*_*i*_ = maximum level of on-hand inventory in units of product *i* when the rework process ends, *t*_*i*_ = time required for producing enough items to meet demand of product *i* during vendor’s uptime *t*_1*i*_ and reworking time *t*_2*i*_, *t*_1*i*_ = production uptime for product *i*, *t*_2*i*_ = the reworking time for product *i*, *t*_3*i*_ = the delivery time for product *i*, *t*_n*i*_ = fixed interval of time between each installment of finished product *i* being delivered during *t*_3i_, *I*(*t*) = level of on-hand perfect quality items at time *t*, *I*_S_(*t*)_*i*_ = level of on-hand scrap items of product *i* at time *t*, *TC*(*Q*_*i*_) = total production-inventory-delivery cost per cycle for product *i*, E[*TCU*(*T*)] = the expected system costs per unit time for *L* products in the proposed system.

From Fig. 1, the following equations (for $i = 1,{ 2}, \ldots ,L$) can be obtained directly:

1 $$T = t_{1i} + t_{2i} + t_{3i} = \frac{{Q_{i} \left[ {1 - \varphi_{i} E\left( {x_{i} } \right)} \right]}}{{\lambda_{i} }}$$

2 $$H_{1i} = \lambda_{i} (t_{1i} + t_{2i} )$$

3 $$H_{2i} = \left( {P_{1i} - d_{1i} } \right)\left( {t_{1i} - t_{i} } \right)$$

4 $$H_{i} = H_{2i} + \left( {P_{2i} - d_{2i} } \right)t_{2i}$$

5 $$t_{i} = \frac{{H_{1i} }}{{P_{1i} - d_{1i} }} = \frac{{\lambda_{i} (t_{1i} + t_{2i} )}}{{P_{1i} - d_{1i} }}$$

6 $$t_{1i} = \frac{{Q_{i} }}{{P_{1i} }} = \frac{{H_{1i} + H_{2i} }}{{P_{1i} - d_{1i} }}$$

7 $$t_{2i} = \frac{{H_{i} - H_{2i} }}{{P_{2i} - d_{2i} }}$$

8 $$t_{3i} = T - \left( {t_{1i} + t_{2i} } \right) = nt_{ni}$$

9 $$\lambda = \sum\limits_{i = 1}^{L} {\lambda_{i} }$$

**Fig. 3:**
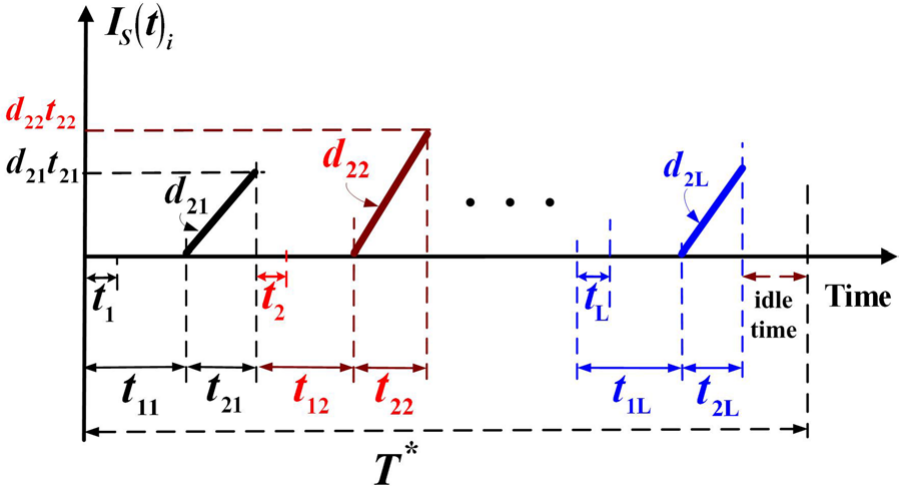
On-hand inventory of scrap items in the proposed multi-item production system with rework failures and a cost reduction distribution policy

The level of on-hand inventory of scrap items in the proposed model is depicted in Fig. 3 and the following equations (for $i = 1,{ 2}, \ldots ,L$) can be obtained:

10 $$t_{2i} = \frac{{x_{i} Q_{i} }}{{P_{2i} }}$$

11 $$d_{1i} t_{1i} = x_{i} Q_{i}$$

The variable holding cost for finished items of product *i* during delivery time *t*_3*i*_ are

12 $$h_{i} \left( {\frac{n - 1}{2n}} \right)H_{i} t_{3i}$$

The fixed and variable transportation costs for product *i* per cycle are

13 $$\left( {n + 1} \right)K_{1i} + C_{{{\text{T}}i}} Q_{i}$$

The production-inventory-delivery cost per cycle for *L* products comprises the setup cost, the variable manufacturing, reworking, and disposal costs (Fig. 3), the fixed and variable shipping cost, the holding cost during *t*_1i_, *t*_2i_, and *t*_3*i*_. Therefore, *TC*(*Q*_i_) becomes

14 $$\sum\limits_{i = 1}^{L} {TC\left( {Q_{i} } \right)} = \sum\limits_{i = 1}^{L} {\left\{ \begin{aligned} K_{i} + C_{i} Q_{i} + C_{{{\text{R}}i}} \left( {x_{i} Q_{i} } \right) + C_{{{\text{S}}i}} \left( {\varphi_{i} x_{i} Q_{i} } \right) + \left( {n + 1} \right)K_{1i} + C_{{{\text{T}}i}} Q_{i} \left( {1 - \varphi_{i} x_{i} } \right) + h_{1i} \left[ {\frac{{d_{1i} t_{1i} }}{2}\left( {t_{2i} } \right)} \right] \hfill \\ + h_{i} \left[ {\frac{{H_{1i} }}{2}\left( {t_{i} } \right) + \frac{{H_{2i} }}{2}\left( {t_{1i} - t_{i} } \right) + \frac{{H_{2i} + H_{i} }}{2}\left( {t_{2i} } \right) + \frac{{d_{1i} t_{1i} }}{2}\left( {t_{1i} } \right) + \left( {\frac{n - 1}{2n}} \right)H_{i} t_{3i} } \right] \hfill \\ \end{aligned} \right\}}$$

Because the defective rate *x* is assumed to be a random variable with a known probability density function, in order to take the randomness of *x* into account, the expected value of *x* is used in this study. By substituting all parameters from Eqs. (1) to (13) in Eq. (14), and with further derivations the expected E[*TCU*(*T*)] can be obtained as follows:

15 $$E\left[ {TCU\left( T \right)} \right] = \sum\limits_{i = 1}^{L} {\left\{ \begin{aligned} &\frac{{C_{i} \lambda_{i} }}{{\left[ {1 - \varphi_{i} E\left[ {x_{i} } \right]} \right]}} + \frac{{K_{i} }}{T} + C_{{{\text{R}}i}} \lambda_{i} \left[ {\frac{{E\left[ {x_{i} } \right]}}{{\left[ {1 - \varphi_{i} E\left[ {x_{i} } \right]} \right]}}} \right] + C_{{{\text{S}}i}} \lambda_{i} \left[ {\frac{{\varphi_{i} E\left[ {x_{i} } \right]}}{{\left[ {1 - \varphi_{i} E\left[ {x_{i} } \right]} \right]}}} \right] + C_{Ti} \lambda_{i} + \frac{{\left( {n + 1} \right)K_{1i} }}{T} \\&+ \frac{{h_{i} T\lambda_{i}^{2} }}{2}\frac{1}{{\left[ {1 - \varphi_{i} E\left[ {x_{i} } \right]} \right]^{2} }}\left\{ \begin{aligned} \lambda_{i} \left[ {\frac{1}{{P_{1i} }} + \frac{{E\left[ {x_{i} } \right]}}{{P_{2i} }}} \right]^{2} \left[ {\frac{{2\lambda_{i} }}{{P_{1i} (1 - E\left[ {x_{i} } \right])}}} \right] - \frac{{E\left[ {x_{i} } \right]}}{{P_{2i} }}\left[ {\frac{1}{{P_{1i} }} - \left[ {1 - E\left[ {x_{i} } \right]} \right]} \right] \hfill \\ + \frac{1}{{P_{1i} }} + \left( {1 - \frac{1}{n}} \right)\left[ {1 - \varphi_{i} E\left[ {x_{i} } \right]} \right]\left[ {\frac{{\left[ {1 - \varphi_{i} E\left[ {x_{i} } \right]} \right]}}{{\lambda_{i} }} - \frac{2}{{P_{1i} }} - \frac{{E\left[ {x_{i} } \right]}}{{P_{2i} }}} \right] \hfill \\ - \left( {1 + \frac{1}{n}} \right)\left[ {\lambda_{i} \left[ {\frac{1}{{P_{1i} }} + \frac{{E\left[ {x_{i} } \right]}}{{P_{2i} }}} \right]^{2} } \right] \hfill \\ \end{aligned} \right\} \\ &\quad \quad\quad+ \frac{{h_{1i} T\lambda_{i}^{2} }}{{2P_{2i} }}\frac{{E\left( {x_{i} } \right)^{2} }}{{\left[ {1 - \varphi_{i} E\left( {x_{i} } \right)} \right]^{2} }} \end{aligned} \right\}}$$

Let

16 $$\begin{aligned} E_{0i} &= \frac{1}{{1 - \varphi_{i} E\left[ {x_{i} } \right]}}; \, E_{1i} = \frac{{E\left[ {x_{i} } \right]}}{{1 - \varphi_{i} E\left[ {x_{i} } \right]}}; \, E_{2i} = \left[ {\frac{1}{{P_{1i} }} + \frac{{E\left[ {x_{i} } \right]}}{{P_{2i} }}} \right]; \\ E_{3i} &= \frac{{2\lambda_{i} }}{{P_{1i} \left[ {1 - E\left[ {x_{i} } \right]} \right]}}; \, E_{4i} = \left[ {1 - \varphi_{i} E\left[ {x_{i} } \right]} \right]\left[ {\frac{{\left[ {1 - \varphi_{i} E\left[ {x_{i} } \right]} \right]}}{{\lambda_{i} }} - \frac{2}{{P_{1i} }} - \frac{{E\left[ {x_{i} } \right]}}{{P_{2i} }}} \right]. \end{aligned}$$

Then Eq. (15) becomes

17 $$E [ {TCU ( T)}] = \sum\limits_{i = 1}^{L} {\left\{ \begin{aligned} C_{i} \lambda_{i} E_{0i} + \frac{{K_{i} }}{T} + C_{{{\text{R}}i}} \lambda_{i} E_{1i} \left[ {\frac{{E\left[ {x_{i} } \right]}}{{\left[ {1 - \varphi_{i} E\left[ {x_{i} } \right]} \right]}}} \right] + C_{{{\text{S}}i}} \lambda_{i} \varphi_{i} E_{1i} + C_{Ti} \lambda_{i} + \frac{{\left( {n + 1} \right)K_{1i} }}{T} + \frac{{h_{1i} T\lambda_{i}^{2} E_{1i}^{2} }}{{2P_{2i} }} \hfill \\ + \frac{{h_{i} T\lambda_{i}^{2} }}{2}\left\{ {\lambda_{i} E_{2i}^{2} E_{3i} - \frac{{E\left[ {x_{i} } \right]}}{{P_{2i} }}\left[ {\frac{1}{{P_{1i} }} - \left[ {1 - E\left[ {x_{i} } \right]} \right]} \right] + \frac{1}{{P_{1i} }} + \left( {1 - \frac{1}{n}} \right)E_{4i} - \left( {1 + \frac{1}{n}} \right)\lambda_{i} E_{2i}^{2} } \right\} \hfill \\ \end{aligned} \right\}}$$

## Derivation of the optimal cycle time

Before derivation of the optimal common production cycle time T*, one should prove that the expected cost function E[*TCU*(*T*)] is convex. By differentiating E[*TCU*(*T*)] with respect to *T* gives the following first and second derivatives:

18 $$\frac{{dE\left[ {TCU(T)} \right]}}{dT} = \sum\limits_{i = 1}^{L} {\left\{ \begin{aligned}& - \frac{{K_{i} }}{{T^{2} }} - \frac{{\left( {n + 1} \right)K_{1i} }}{{T^{2} }} + \frac{{h_{1i} \lambda_{i}^{2} E_{1i}^{2} }}{{2P_{2i} }} \\ &+ \frac{{h_{i} \lambda_{i}^{2} }}{2}\left\{ {\lambda_{i} E_{2i}^{2} E_{3i} - \frac{{E\left[ {x_{i} } \right]}}{{P_{2i} }}\left[ {\frac{1}{{P_{1i} }} - \left[ {1 - E\left[ {x_{i} } \right]} \right]} \right] + \frac{1}{{P_{1i} }} + \left( {1 - \frac{1}{n}} \right)E_{4i} - \left( {1 + \frac{1}{n}} \right)\lambda_{i} E_{2i}^{2} } \right\} \hfill \\ \end{aligned} \right\}}$$

19 $$\frac{{d^{2} E\left[ {TCU(T)} \right]}}{{dT^{2} }} = \sum\limits_{i = 1}^{L} {\left\{ {\frac{{2\left[ {K_{i} + \left( {n + 1} \right)K_{1i} } \right]}}{{T^{3} }}} \right\}}$$

It can be seen that Eq. (19) is positive, since *K*_i_*, n, K*_1i_, and *T* are all positive. Because the second derivative of *E[TCU(T)]* > 0, one confirms that *E[TCU(T)]* is convex for all *T* different from zero. It follows that by letting the first derivative of *E[TCU(T)]* = 0, one can derive the optimal common production cycle time T*. Let

20 $$\frac{{dE\left[ {TCU(T)} \right]}}{dT} = \sum\limits_{i = 1}^{L} {\left\{ \begin{aligned} &- \frac{{K_{i} }}{{T^{2} }} - \frac{{\left( {n + 1} \right)K_{1i} }}{{T^{2} }} + \frac{{h_{1i} \lambda_{i}^{2} E_{1i}^{2} }}{{2P_{2i} }} \\ &+ \frac{{h_{i} \lambda_{i}^{2} }}{2}\left\{ {\lambda_{i} E_{2i}^{2} E_{3i} - \frac{{E\left[ {x_{i} } \right]}}{{P_{2i} }}\left[ {\frac{1}{{P_{1i} }} - \left[ {1 - E\left[ {x_{i} } \right]} \right]} \right] + \frac{1}{{P_{1i} }} + \left( {1 - \frac{1}{n}} \right)E_{4i} - \left( {1 + \frac{1}{n}} \right)\lambda_{i} E_{2i}^{2} } \right\} \end{aligned} \right\}} = 0$$

or

21 $$\frac{1}{{T^{2} }}\sum\limits_{i = 1}^{L} {\left[ {K_{i} + \left( {n + 1} \right)K_{1i} } \right]} = \sum\limits_{i = 1}^{L} {\lambda_{i}^{2} \left\{ {\frac{{h_{1i} E_{1i}^{2} }}{{2P_{2i} }} + \frac{{h_{i} }}{2}\left[ \begin{aligned} \lambda_{i} E_{2i}^{2} E_{3i} - \frac{{E\left[ {x_{i} } \right]}}{{P_{2i} }}\left[ {\frac{1}{{P_{1i} }} - \left[ {1 - E\left[ {x_{i} } \right]} \right]} \right] \hfill \\ + \frac{1}{{P_{1i} }} + \left( {1 - \frac{1}{n}} \right)E_{4i} - \left( {1 + \frac{1}{n}} \right)\lambda_{i} E_{2i}^{2} \hfill \\ \end{aligned} \right]} \right\}}$$

Therefore, one has T* as follows:

22 $$T^{*} = \sqrt {\frac{{2\sum\limits_{i = 1}^{L} {\left[ {K_{i} + \left( {n + 1} \right)K_{1i} } \right]} }}{{\sum\limits_{i = 1}^{L} {\lambda_{i}^{2} \left\{ {\frac{{h_{1i} E_{1i}^{2} }}{{P_{2i} }} + h_{i} \left[ {\lambda_{i} E_{2i}^{2} E_{3i} - \frac{{E\left[ {x_{i} } \right]}}{{P_{2i} }}\left[ {\frac{1}{{P_{1i} }} - \left[ {1 - E\left[ {x_{i} } \right]} \right]} \right] + \frac{1}{{P_{1i} }} + \left( {1 - \frac{1}{n}} \right)E_{4i} - \left( {1 + \frac{1}{n}} \right)\lambda_{i} E_{2i}^{2} } \right]} \right\}} }}}$$

### Capacity and setup time effects on the optimal cycle time

Generally speaking, setup time is relatively short in comparison with uptime. But, if the setup time becomes a factor, one has to ensure that the cycle length is long enough to account for the setup, production, and reworking times of *L* products (Nahmias 2009). Let *S*_*i*_ denote the production setup time for product *i*, the Eq. (23) must hold.

23 $$\sum\nolimits_{i = 1}^{L} {\left[ {S_{i} + \left( {Q_{i} /P_{1i} } \right) + \left( {x_{i} Q_{i} /P_{2i} } \right)} \right]} { < }T$$

Substituting Eq. (1) in Eq. (23) one has

24 $$T > \frac{{\sum\nolimits_{i = 1}^{L} {S_{i} } }}{{1 - \sum\nolimits_{i = 1}^{L} {\left( {\left( {\lambda_{i} /P_{1i} } \right) + \left( {x_{i} \lambda_{i} /P_{2i} } \right)} \right)} }} = T_{\hbox{min} .}$$

Therefore, when setup time becomes a significant factor in production planning, one must choose the optimal common cycle length from the maximum of [T*, *T*_min_].

## Numerical example

With the purpose of easing the comparison efforts for readers, this section uses the same example as in Chiu et al. (2013) to demonstrate the proposed research result. Reconsider a production plan of producing five different items on a single machine in sequence under a common cycle time policy. Annual demand rates *λ*_*i*_ for these five items are 3000, 3200, 3400, 3600, and 3800, respectively. The production rates *P*_1i_ are 58,000; 59,000; 60,000; 61,000 and 62,000, respectively. During individual production process, there are random nonconforming rates *x*_*i*_ for each item and they follow Uniform distribution over the intervals of [0, 0.05], [0, 010], [0, 0.15], [0, 020], and [0, 0.25], respectively. All nonconforming items produced go through a rework process at the rates *P*_2i_ of 1800, 2000, 2200, 2400, and 2600 items per year, respectively; with additional unit rework costs *C*_R*i*_ of $50, $55, $60, $65, and $70, respectively. During the reworking, there are failure-in-rework rates *φ*_i_ of 0.05, 0.10, 0.15, 0.20, and 0.25, respectively. Additional values of system parameters are given as follows: *K*_*i*_ = the setup costs are $3800, $3900, $4000, $4100, and $4200, respectively, *C*_*i*_ = production costs per item are $80, $90, $100, $110, and $120, respectively, *C*_S*i*_ = disposal costs per item are $20, $25, $30, $35, and $40, respectively, *K*_1*i*_ = fixed costs per delivery are $1800, $1900, $2000, $2100, and $2200, respectively, *C*_T*i*_ = unit transportation costs are $0.1, $0.2, $0.3, $0.4, and $0.5, respectively, *n* = number of shipments per cycle, it is assumed to be a constant 3 (i.e., *n* + 1 = 4), *h*_*i*_ = unit holding costs are $10, $15, $20, $25, and $30, respectively, *h*_1*i*_ = holding costs per reworked item are $30, $35, $40, $45, and $50, respectively.

**Fig. 4:**
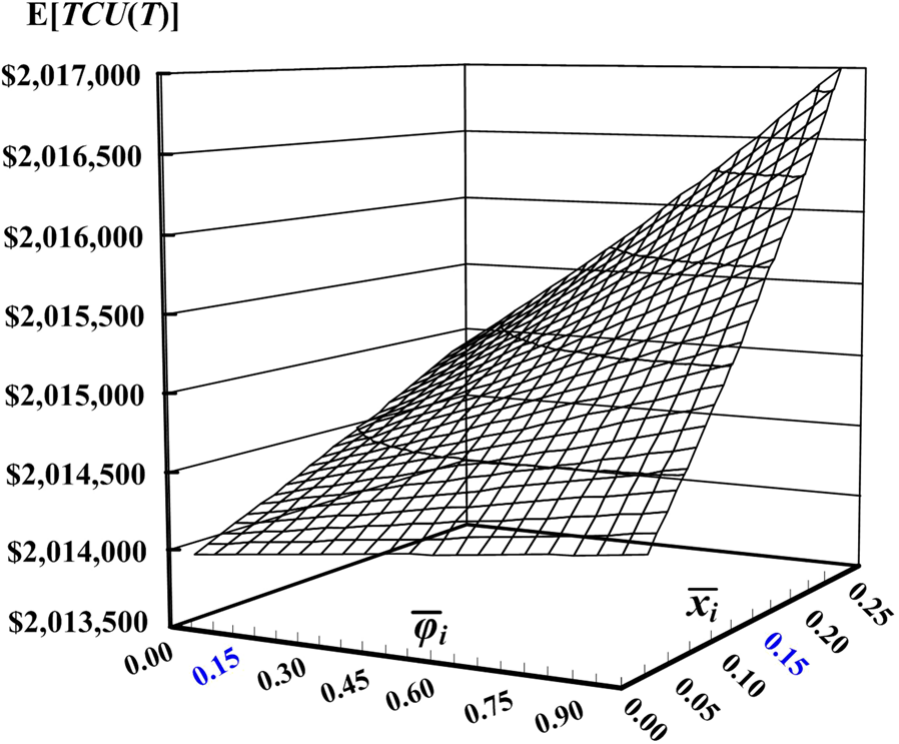
Variation of mean defective rate and mean failure-in-rework rate effects on the expected system cost E[*TCU*(*T*)]

The optimal common cycle time T* = 0.7279 (years) can be obtained by applying Eq. (22). Total expected system costs E[*TCU*(T*)] = $2,013,956 can also be obtained from computation of Eq. (15). Variation of mean defective rate and mean failure-in-rework rate effects on the expected system cost E[*TCU*(*T*)] is illustrated in Fig. 4. It is noted that as mean defective rate increases the E[*TCU*(*T*)] increases significantly, and as mean failure-in-rework rate increases the system cost E[*TCU*(*T*)] increases slightly.

**Fig. 5:**
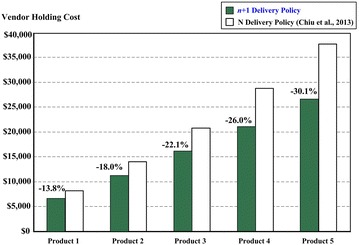
Percentage of reduction in producer’s inventory holding costs for five different products in example, respectively

As stated earlier, the proposed model aims at reducing vendor’s inventory holding cost for each product *i* during the production cycle. As a result from this numerical example, the percentage of overall holding cost reduction is 24.7 % (i.e., from $109,476 (Chiu et al. 2013) down to $82,431). Figure 5 demonstrates the percentage of holding cost reduction for five different products, respectively as compared to that of Chiu et al.’s work (where *n*-delivery policy is adopted).

In summary, the proposed study realizes a significant system cost savings of $56,358 (i.e., $2,070,314 − $2,013,956) or 16.09 % of other system interrelated costs (i.e., E[*TCU*(*T*)] − *λC*, which is the expected system cost excludes variable manufacturing cost).

## Conclusions

With an aim at reducing producer’s inventory cost as well as minimizing the expected overall system costs per unit time, this study incorporating an enhanced *n* + 1 product issuing policy into Chiu et al.’s model (2013), and with the help of mathematical modeling and optimization method a closed-form optimal common production cycle time for the proposed multi-product EMQ model with rework failures was derived. A numerical example is given to demonstrate the applicability of our research result, reveal joint effects of random defective rate and failure-in-rework rate on the optimal policy (refer to Fig. 4), and confirm significant savings in producer’s inventory holding cost (Fig. 5). For future study, an interesting topic will be to include the machine breakdown factor into such a multi-product EMQ model.
